# Statin use is associated with reduced mortality after respiratory viral infection

**DOI:** 10.1183/23120541.00365-2020

**Published:** 2021-02-01

**Authors:** Juan Antonio Franco-Peláez, Laura Esteban-Lucia, María de los Ángeles Zambrano Chacón, Ana María Pello-Lázaro, Ana María Venegas Rodriguez, Luis Nieto Roca, Camila Sofia García-Talavera, Andrea Kallmeyer Mayor, Felipe Villar Alvarez, Ricardo Fernandez Roblas, Oscar Gonzalez-Lorenzo, José Tuñón, Borja Ibañez, Alvaro Aceña

**Affiliations:** 1 Dept of Cardiology, Instituto de Investigación Sanitaria-Fundación Jiménez Díaz, Madrid, Spain; 2 Dept of Pneumology, Instituto de Investigación Sanitaria -Fundación Jiménez Díaz, Madrid, Spain; 3 Autonoma University, Madrid, Spain; 4 CIBERES, Madrid, Spain; 5 Dept of Microbiology, Instituto de Investigación Sanitaria-Fundación Jiménez Díaz, Madrid, Spain; 6 CIBERCV, Madrid, Spain; 7 Centro Nacional de Investigaciones Cardiovasculares (CNIC), Madrid, Spain

## Abstract

**Background:**

Several studies suggest that statins, besides reducing cardiovascular disease, have anti-inflammatory properties which might provide a benefit in downregulating the immune response after a respiratory viral infection (RVI) and, hence, decreasing subsequent complications. We aim to analyse the effect of statins on mortality after RVI.

**Methods:**

A single-centre, observational and retrospective study was carried out including all adult patients with a RVI confirmed by PCR tests from October 2, 2017 to May 20, 2018. Patients were divided between statin users and non-statin users and followed-up for 1 year, and all causes of death were recorded. In order to analyse the effect of statin treatment on mortality after RVI we planned two different approaches, a multivariate Cox regression model with the overall population and a univariate Cox model with a propensity-score matched population.

**Results:**

We included 448 patients, 154 (34.4%) of whom were under statin treatment. Statin users had a worse clinical profile (older population with more comorbidities). During the 1-year follow-up, 67 patients died, 17 (11.0%) in the statin group and 50 (17.1%) in the non-statin group. Multivariate Cox analysis showed that statins were associated with mortality benefit (HR 0.47, 95% CI 0.26–0.83; p=0.01). In a matched population (101 statins users and 101 non-statins users) statins also remained associated with mortality benefit (HR 0.32, 95% CI 0.14–0.72; p=0.006). Differences were mainly driven by non-cardiovascular mortality (HR 0.31, 95% CI 0.13–0.73; p=0.004).

**Conclusions:**

Chronic statin treatment was associated with reduced 1-year mortality in patients with laboratory-confirmed RVI. Further studies are needed to determine the exact role of statin therapy after RVI.

## Abstract 2

**Statin treatment is associated with reduced 1-year mortality after respiratory viral infections, despite the higher risk profile of patients on statins. Statins seem a good candidate to be tested during the current global pandemic.** https://bit.ly/36t0tDh

## Introduction

Acute respiratory infections are the leading cause of morbidity and one of the principal causes of mortality worldwide. Viruses are responsible for 53% of reported cases, and when hospital admission is required, they become a serious health problem [1]. Recent studies have highlighted that in association with respiratory damage, there is an increased risk of developing a cardiovascular event following the infectious process [2].

Within the first days after an influenza infection [2–4], respiratory syncytial virus and other respiratory viral infections (RVI) [3, 5], patients have a six-fold increased risk of acute myocardial infarction. Likewise, myocardial infarction occurs in cases of bacterial infection such as pneumococcal pneumonia (7–8% higher risk of myocardial infarction reported [6, 7]), *Haemophilus influenzae* pneumonia [8], bacteraemias [5] and urinary tract infections [9].

The pathophysiological mechanism proposed is complex, but predominantly RVI results in a powerful and sustained release of inflammatory mediators [2, 10]. A considerable proportion of patients who have recovered from a RVI show higher values of pro-inflammatory molecules [11], such as C-reactive protein, methyl-accepting chemotaxis proteins (MCP) or interleukin-6, which suggests the presence of sustained inflammation is associated with a significant increase in the risk of developing major adverse events, including death [12].

Currently there is no therapeutic option to reduce the post-RVI adverse clinical events [13]. However, in view of the fact that cholesterol allows invasion by pathogens by acting as a docking site for the internalisation of virus [14], treatment with statins has been considered for these patients. This hypothesis was initially suggested by Fedson *et al.* [15] as a treatment to reduce mortality in the 2003 H5N1 pandemic.

Large clinical trials have shown evidence that statins reduce major adverse events by 35%. This benefit seems to be driven not only by reduction in cholesterol levels, but also by the so-called “pleiotropic effects”. The latter include a potential immunomodulatory and anti-inflammatory effect of statins [14, 16]. That is why according to the hypolipaemic, immunomodulatory and anti-inflammatory effects of statins, this treatment could provide protection to patients who have suffered an RVI.

Few studies have investigated treatment with statins after infection. A beneficial effect has been demonstrated by reducing levels of biomarkers related to increased cardiovascular risk, such as IL-6 [17], MCP-1 [17] or C-reactive protein [18], and they seem to be useful in infections with a significant immune system response [19], such as bacterial sepsis. However, regarding viral respiratory disorders, studies have led to controversial conclusions, some of them showing mortality reduction in patients on statins after 30 days of follow-up [13], and others showing no effect within 90 days of follow-up [20]. This controversy remains when considering other respiratory infections. Frost *et al.* [19] found a reduced risk of death from COPD among statin users and a significantly reduced risk of death from influenza/pneumonia, while Majumdar *et al.* [21] reported no benefit in mortality reduction or intensive care unit admission in patients with pneumonia.

Taking into account those controversies and the current situation with the coronavirus disease 2019 pandemic, where it has been shown that the pro-inflammatory effect plays a key role in the evolution of the disease [22], we aim to assess the prognostic effect of statin treatment in patients that have suffered an RVI.

## Methods

### Patients and study design

We included all consecutive patients older than 18 years who had been diagnosed with RVI after confirmation by PCR on nasopharyngeal swabs at the Microbiology Department of our institution during the 2017–2018 Comprehensive Influenza Surveillance Period (established by the Carlos III National Institute Epidemiology Center from October 2, 2017 to May 20, 2018). Baseline characteristics and current prescribed drugs were recorded from electronic health records. Patients were divided into two groups, statin users and non-statin users, and data from follow-up 1 year after the date of the positive PCR test were taken from electronic health records, specifying vital status and, in case of death, its cause (cardiovascular or non-cardiovascular). The primary end-point of our study was all-cause death and the secondary end-points were cardiovascular and non-cardiovascular death. Cardiovascular death was defined as mortality related to myocardial infarction, heart failure or stroke.

### Statistical analysis

Quantitative data are presented as median and interquartile range. Comparisons between quantitative variables were performed with t-test or Mann–Whitney test where appropriate. Qualitative variables are shown as frequencies and percentages and were compared using Chi-squared or Fisher's exact test where appropriate.

In order to analyse the effect of statin treatment on mortality after an RVI we planned two different approaches, a multivariate proportional hazards Cox regression model with the overall population and a univariate Cox regression model with a propensity-score matched population. To carry out the multivariate Cox model, we first performed a univariate analysis including all clinically relevant variables, and those with a p-value <0.20 remained in the multivariate analysis, which was made using a backward stepwise method. We considered independent predictors as those variables with a p-value <0.05 after this analysis. The propensity-score matched population was selected after performing binary logistic regression analysis, taking statin treatment as the dependent variable and age, sex, hypertension, diabetes, smoking history, previous stroke, peripheral arterial disease and ischaemic heart disease as independent variables. With the estimated probability of statin treatment, we matched both groups, in a 1:1 ratio, with the nearest neighbour method (caliper=0.2×sd[logitPs]). Finally, matched groups were compared with univariate Cox analysis, and survival curves were drawn using the Kaplan–Meier method.

All statistical analysis was performed with IBM SPSS Statistics for Windows, Version 20.0 (IBM Corp, Armonk, NY, USA).

### Ethics statement

The research protocol complies with the Declaration of Helsinki and was approved by the ethics committee of our institution (EO012-20 FJD).

## Results

We included 448 patients who met the inclusion criteria; 154 of them (34.4%) were on statin treatment and 294 (65.6%) were not. Baseline characteristics are shown in table 1. It should be noted that there were several differences between the groups: statin users were older, more frequently male, had a higher prevalence of hypertension, diabetes, coronary artery disease, heart failure, peripheral artery disease or cerebrovascular disease, and had a lower glomerular filtration rate. Furthermore, the proportion of patients vaccinated against flu was higher among those on statins than those that were not.

**TABLE 1:** Baseline characteristics of statin and non-statin users and overall cohort

	**Overall cohort**	**Non-statin users**	**Statin users**	**p-value**
**Subjects n**	448	294	154	
**Age years**	73.5 (60–84)	68 (54–83)	79 (71–85)	**<0.001**
**Female sex**	234 (52.2)	167 (56.8)	67 (43.5)	**0.007**
**Hypertension**	247 (55.1)	125 (42.5)	122 (79.2)	**<0.001**
**Diabetes mellitus**	97 (21.7)	36 (12.2)	61 (39.6)	**<0.001**
**Current smoker**	73 (16.3)	53 (18.0)	20 (13.0)	0.16
**CAD**	46 (10.3)	5 (1.7)	41 (26.6)	**<0.001**
**Heart failure**	53 (11.8)	19 (6.5)	34 (22.1)	**<0.001**
**PAD**	35 (7.8)	7 (2.4)	28 (18.2)	**<0.001**
**CVD**	24 (5.4)	7 (2.4)	17 (11.0)	**<0.001**
**COPD**	81 (18.1)	54 (18.4)	27 (17.5)	0.83
**Cancer**	122 (27.2)	83 (28.2)	39 (25.3)	0.51
**Influenza vaccination**	185 (42.9)	96 (33.9)	89 (60.1)	**<0.001**
**Haemoglobin g·dL^−1^**	13.0 (11.8–14.0)	13.0 (11.8–14.1)	13.0 (11.7–14.0)	0.43
**Platelets ×10^3^ cells·µL^−1^**	204 (152–259)	207 (155–267)	195 (150–234)	0.15
**Leukocytes ×10^3^ cells·µL^−1^**	7.9 (5.5–10.6)	7.7 (5.0–10.2)	8.1 (6.0–11.6)	0.81
**Neutrophils ×10^3^ cells·µL^−1^**	6.0 (3.7–8.9)	5.8 (3.3–8.6)	6.6 (4.5–9.3)	0.41
**GFR mL·min^−1^ per 1.73 m^2^**	80 (60–96)	86 (68–101)	72 (55–84)	**<0.001**
**CRP peak mg·dL^−1^**	5.8 (2.5–17.1)	5.8 (2.4–16.9)	6.0 (2.7–18.0)	0.76

Data are presented as median (interquartile range) or n (%), unless otherwise stated. CAD: coronary artery disease; PAD: peripheral artery disease; CVD: cerebrovascular disease; GFR: glomerular filtration rate; CRP: C-reactive protein. p-value denotes comparison between statin and non-statin users. Bold indicates statistical significance.

Regarding the type of virus, influenza viruses (A or B) were responsible for 41.3% of cases of RVI, far higher than cases due to rhinovirus (19.6%), respiratory syncytial virus (14.3%) and metapneumovirus (12.7%). There were no significant differences in the type of virus detected between the study groups (table 2). Biological samples were collected from hospital wards (55.1%), emergency departments (43.1%) and primary care facilities (1.8%).

**TABLE 2:** Type of virus detected in nasopharyngeal swabs and responsible for respiratory viral infection

**Virus type**	**Overall cohort**	**Non-statin users**	**Statin users**
**Subjects n**	448	294	154
**Influenza virus**	185 (41.5)	127 (43.2)	58 (37.7)
Influenza A	115 (25.7)	76 (25.9)	39 (25.3)
Influenza B	70 (15.6)	51 (17.3)	19 (12.3)
**Rhinovirus**	88 (19.6)	62 (21.1)	26 (16.9)
**Respiratory syncytial virus**	64 (14.3)	38 (12.9)	26 (16.9)
**Metapneumovirus**	57 (12.7)	33 (11.2)	24 (15.6)
**Coronavirus**	21 (4.7)	14 (4.8)	7 (4.5)
**Human parainfluenza**	19 (4.2)	7 (2.4)	12 (7.8)
**Adenovirus**	1 (0.2)	1 (0.3)	0 (0.0)
**Multiple viruses**	13 (2.9)	12 (4.1)	1 (0.6)

Data are presented as n (%) unless otherwise stated.

During the first-year follow-up period after RVI, 67 patients died (15.0%): 50 (17.1%) in the non-statin and 17 (11.0%) in the statin group. Non-cardiovascular death was attributed to 63 patients (16 statin and 47 non-statin users) and cardiovascular death to three patients (one statin and two non-statin users).

**FIGURE 1:**
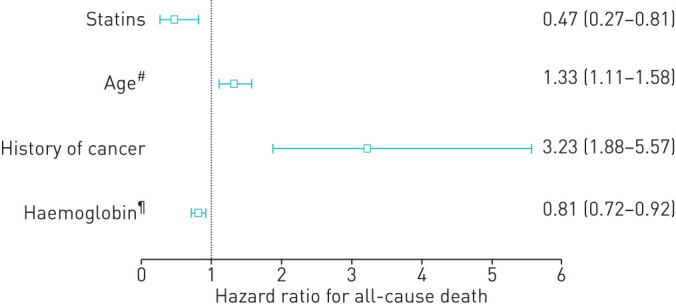
Independent predictors of all-cause death after multivariate proportional hazards Cox regression analysis in the overall population. ^#^: for every 10 years; ^¶^: for every gramme per decilitre.

At univariate Cox analysis, variables which met the criterion of p<0.20 were age (0.029), sex (0.072), cancer (<0.001), influenza infection (0.168), haemoglobin (<0.001), platelet count (0.066), C-reactive protein peak (0.028) and angiotensin-converting enzyme inhibitors or angiotensin receptor blockers prescription (0.132), in addition to statin intake (0.040). Multivariate Cox regression showed that statin therapy was an independent predictor of mortality (hazard ratio (HR) 0.47, 95% CI 0.27–0.81, p=0.005), along with age (HR 1.33 per 10 years, 95% CI 1.11–1.58, p=0.001), history of cancer (HR 3.23, 95% CI 1.88–5.57, p<0.001) and haemoglobin level (HR 0.81 for every gramme per decilitre, 95% CI 0.72–0.92, p=0.001) (figure 1).

**FIGURE 2:**
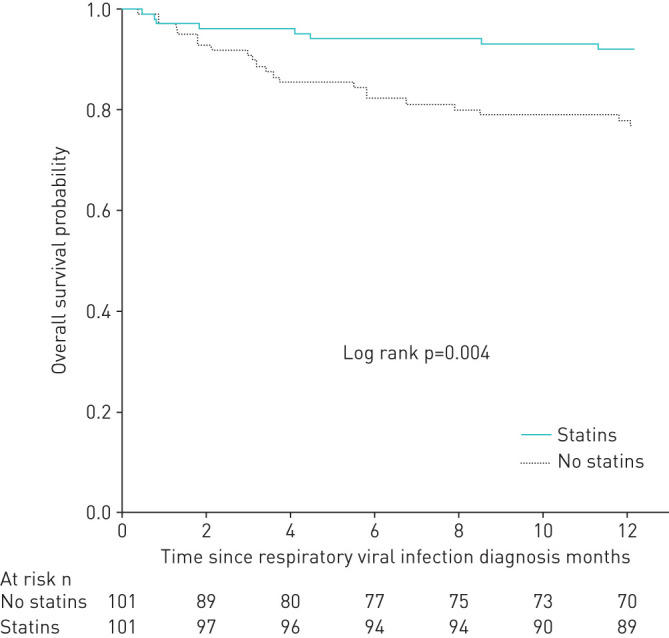
Kaplan–Meier survival curves showing the differences in all-cause death in matched population. Continuous line represents statin users. Dashed line represents non-statin users.

**FIGURE 3:**
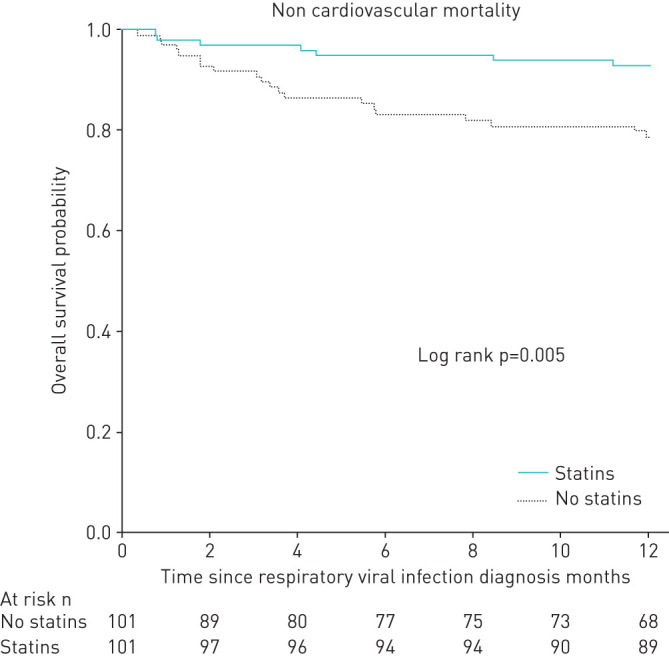
Kaplan–Meier survival curves showing the differences in non-cardiovascular death in matched population. Continuous line represents statin users. Dashed line represents non-statin users.

**FIGURE 4:**
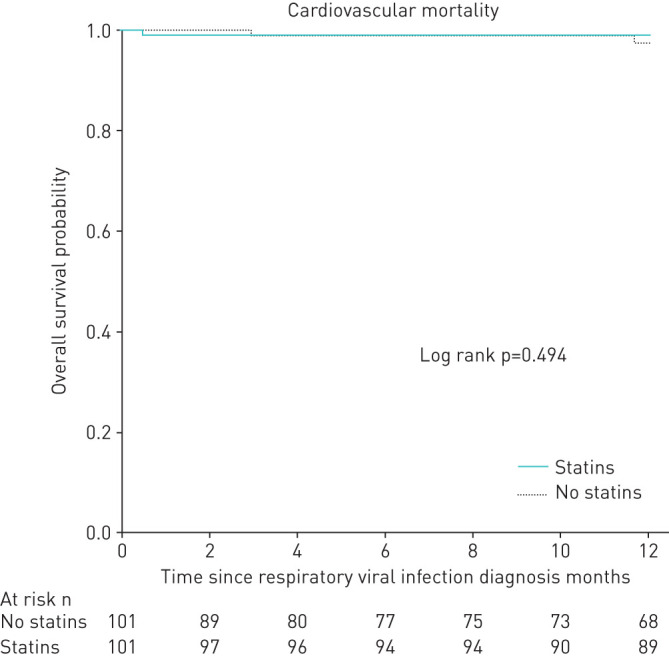
Kaplan–Meier survival curves showing the differences in cardiovascular death in matched population. Continuous line represents statin users. Dashed line represents non-statin users.

After propensity-score matching, 101 statin patients were matched with 101 control patients. Baseline characteristics can be seen in table 3. Both groups had well balanced baseline characteristics except for heart failure prevalence (24% in statin and 9% in non-statin groups, respectively, p=0.004). There were 30 deaths (two cardiovascular and 28 non-cardiovascular), eight (7.9%) in the statin group and 22 (21.8%) in the non-statin group. Cox analysis showed that statin use was associated with less all-cause death during follow-up with a HR 0.32 (95% CI 0.14–0.72; p=0.006). Differences in mortality were mainly driven by non-cardiovascular mortality (HR 0.31, 95% CI 0.13–0.73; p=0.004), whereas there were no differences in cardiovascular mortality (HR 0.44, 95% CI 0.04–4.88; p=0.49). Figure 2 shows Kaplan–Meier survival curves of all-cause death. Kaplan–Meier survival curves of non-cardiovascular death are shown in figure 3, and cardiovascular death is represented in figure 4.

**TABLE 3:** Baseline characteristics of the propensity-score matched population (statin and non-statin users)

	**Non-statin users**	**Statin users**	**p-value**
**Subjects n**	101	101	
**Age years**	80 (69.5–88.0)	79 (70.5–84.0)	0.284
**Female sex**	47 (46.5)	54 (53.5)	0.325
**Hypertension**	81 (80.2)	76 (75.2)	0.398
**Diabetes mellitus**	25 (24.8)	29 (28.7)	0.525
**Current smoker**	13 (12.9)	12 (11.9)	0.831
**CAD**	5 (5.0)	6 (5.9)	0.757
**Heart failure**	9 (8.9)	24 (23.8)	**0.004**
**PAD**	7 (6.9)	6 (5.9)	0.774
**CVD**	7 (6.9)	8 (7.9)	0.788
**COPD**	18 (17.8)	15 (14.9)	0.568
**Cancer**	29 (28.7)	24 (23.8)	0.424
**Influenza vaccination**	48 (48.5)	61 (63.5)	0.105
**Haemoglobin g·dL^−1^**	13.0 (11.9–13.9)	13.0 (12.2–14.0)	0.523
**Platelets ×10^3^ cells·µL^−1^**	207 (140.0–266.0)	196 (154.5–250)	0.600
**Leukocytes ×10^3^ cells·µL^−1^**	8.0 (5.3–11.0)	8.0 (6.1–11.9)	0.476
**Neutrophils ×10^3^ cells·µL^−1^**	6.2 (3.6–9.2)	6.5 (4.6–9.9)	0.264
**GFR mL·min^−1^ per 1.73 m^2^**	77.9 (57.6–91.4)	77.1 (56.0–85.1)	0.208
**CRP peak mg·dL^−1^**	5.8 (2.6–17.6)	5.0 (2.7–20.0)	0.726

Data are presented as median (interquartile range) or n (%), unless otherwise stated. CAD: coronary artery disease; PAD: peripheral artery disease; CVD: cerebrovascular disease; GFR: glomerular filtration rate; CRP: C-reactive protein. p-value denotes comparison between statin and non-statin users. Bold indicates statistical significance.

## Discussion

The main finding of our study is that chronic statin treatment is associated with reduced 1-year mortality in patients with PCR-confirmed RVI, even though patients on statin had a significantly higher risk profile (older, more frequently male, with a higher prevalence of hypertension, diabetes, ischaemic heart disease, heart failure, peripheral artery disease and cerebrovascular disease, and with a lower glomerular filtration rate). In spite of our modest sample size (n=448), the high event rate observed in our study (67 deaths) reinforces the Cox regression findings, as the ratio of number of events to number of independent predictors is around 17 and above the level suggested by Peduzzi and colleagues [23, 24]. Furthermore, the results were confirmed with the propensity-score matching approach, which is another well-known way to avoid confounding factors in observational studies [25].

Statins are widely recommended for treatment of hyperlipidaemia and cardiovascular diseases and for prevention of ischaemic heart disease and stroke. However, when analysing the causes of death among patients on statin treatment during recovery from RVI, we found that the reduction of mortality was clearly driven by non-cardiovascular causes, therefore suggesting a new pleiotropic effect far beyond the well-known cardiovascular protective properties of statins. Nevertheless, we cannot exclude a beneficial effect on cardiovascular mortality as the number of deaths in our study was low (only three patients), but the number involved was not large enough to show significant differences between the groups, and larger studies are required to assess whether those differences exist or not.

Moreover, other therapeutic effects of statins have emerged recently. Some studies have shown that statin treatment is associated with lower cancer-related mortality rates [26], *e.g.* lung cancer [27] and hepatocellular carcinoma [28]. Furthermore, there is a negative association between statin therapy and the risk of hepatocellular carcinoma development in patients with viral hepatitis B [29], which, like our study, suggests an immunomodulatory and anti-inflammatory effect of statins on the viral trigger.

A meta-analysis of five randomised clinical trials has shown that people vaccinated against influenza viruses have a 36% lower risk of cardiovascular events [30]. We also found in our study that the statin users, who were older and had a significantly higher prevalence of cardiovascular risk factors, were more likely to have had previous influenza vaccination. However, interaction of statin treatment with age and flu vaccination was ruled out.

Concerning the effect of statins on viral respiratory infections, Vandermeer *et al.* [13] showed a beneficial effect at 30 days follow-up while Atamna *et al.* [20] showed no benefits at 90 days follow-up. In our study, we have observed that the benefit did not appear in the first month as described by Vandermeer *et al.* [13] but instead during the long-term follow-up (unlike Atamna *et al*.’s results). This raises the possibility that statins might also have a mortality benefit if administered during the respiratory infection episode.

Most studies with statins in respiratory infections involve influenza viruses or bacterial pneumonia. However, our study included different types of viruses, including coronavirus, which allowed us to assess the effect of statin treatment on infections caused by different viruses, without being limited to influenza infections. Therefore, given the current global coronavirus disease 2019 pandemic, statins appear to be good candidates for prospective randomised clinical trials recruiting patients discharged after viral infection.

Some limitations must be taken into account. First, this is an observational study, and the information was obtained by reviewing electronic health records of patients. In addition, it is a single-centre study, and the patients selected were those with RVI confirmed by laboratory tests, so the study might not be fully representative of the whole population. Although we recorded the data from drug prescriptions, we were not able to ensure patients’ treatment compliance, which could be another limitation. Finally, the number of cardiovascular deaths in our study was not enough to find significant differences between the groups. Studies with larger sample sizes would be needed to assess whether real differences exist.

### Conclusion

Our study findings suggest that chronic statin treatment is associated with reduced 1-year mortality in patients with a laboratory-confirmed RVI. This reduction in mortality was clearly driven by non-cardiovascular deaths (suggesting an important pleiotropic effect) and was maintained when comparing patients through propensity-score matching. Thus, statins seem to be a good candidate for pharmacological treatment of patients discharged after a RVI in a prospective randomised clinical trial.
